# A Gradual Process of Recombination Restriction in the Evolutionary History of the Sex Chromosomes in Dioecious Plants

**DOI:** 10.1371/journal.pbio.0030004

**Published:** 2004-12-21

**Authors:** Michael Nicolas, Gabriel Marais, Vladka Hykelova, Bohuslav Janousek, Valérie Laporte, Boris Vyskot, Dominique Mouchiroud, Ioan Negrutiu, Deborah Charlesworth, Françoise Monéger

**Affiliations:** **1**Laboratoire de Reproduction et Développement des Plantes, ENS LyonLyonFrance; **2**Institute of Evolutionary Biology, School of Biological ScienceUniversity of Edinburgh, King's Buildings, West Mains Road, EdinburghUnited Kingdom; **3**Laboratory of Plant Developmental Genetics, Institute of BiophysicsAcademy of Sciences of the Czech Republic, BrnoCzech Republic; **4**Laboratoire de Biométrie et Biologie Evolutive, Bâtiment Gregor MendelVilleurbanne CedexFrance; University of UppsalaSweden

## Abstract

To help understand the evolution of suppressed recombination between sex chromosomes, and its consequences for evolution of the sequences of Y-linked genes, we have studied four X-Y gene pairs, including one gene not previously characterized, in plants in a group of closely related dioecious species of *Silene* which have an X-Y sex-determining system *(S. latifolia, S. dioica,* and *S. diclinis).* We used the X-linked copies to build a genetic map of the X chromosomes, with a marker in the pseudoautosomal region (PAR) to orient the map. The map covers a large part of the X chromosomes—at least 50 centimorgans. Except for a recent rearrangement in *S. dioica,* the gene order is the same in the X chromosomes of all three species. Silent site divergence between the DNA sequences of the X and Y copies of the different genes increases with the genes' distances from the PAR, suggesting progressive restriction of recombination between the X and Y chromosomes. This was confirmed by phylogenetic analyses of the four genes, which also revealed that the least-diverged X-Y pair could have ceased recombining independently in the dioecious species after their split. Analysis of amino acid replacements vs. synonymous changes showed that, with one possible exception, the Y-linked copies appear to be functional in all three species, but there are nevertheless some signs of degenerative processes affecting the genes that have been Y-linked for the longest times. Although the X-Y system evolved quite recently in *Silene* (less than 10 million years ago) compared to mammals (about 320 million years ago), our results suggest that similar processes have been at work in the evolution of sex chromosomes in plants and mammals, and shed some light on the molecular mechanisms suppressing recombination between X and Y chromosomes.

## Abstract 2

Similar processes have been at work in the evolution of sex chromosomes in plants and mammals. A recently evolved plant X-Y system is helping to shed light on these processes

## Introduction

Newly evolved sex chromosome systems, such as those in plants [1] and fish [2] allow study of the evolutionary processes causing degeneration of Y chromosomes. The genetic theory of sex chromosome evolution [3] predicts that initially one part of a chromosome pair containing the sex-determining genes evolves reduced recombination. Two questions are then particularly interesting. First, how is recombination suppressed throughout most of the initially homologous X and Y chromosomes, as in mammalian and *Drosophila* sex chromosomes and some plants [1], but not others [4]? Second, why does recombination suppression lead to genetic degeneration? Processes leading to degeneration in large nonrecombining genome regions have been well studied theoretically [5], and empirical data on the first stages of degeneration are starting to be obtained from the plant genus *Silene* [6,7] and from the neo-sex chromosomes of Drosophila miranda [8]. Recent neo-sex chromosome systems in *Drosophila* are excellent for studying the rate and causes of degeneration, but cannot shed light on question (i).

Studies of the evolutionary divergence of gene pairs on mammalian X and Y chromosomes suggest that recombination between the X and nonrecombining parts of the Y was successively suppressed. In many X-Y systems, including that in mammals, there is a “pseudoautosomal” region (PAR) where the X and Y recombine, and it has been found that DNA sequence divergence between homologous X- and Y-linked genes increases with distance from this region. This pattern has been termed “evolutionary strata” [9,10]. Part of the reason for different sequence divergence is that mammalian sex chromosomes are ancient neo-sex chromosomes [11]. In addition, the “strata” suggest a series of Y inversions disrupting X-Y recombination [9]. Strata have also been found in the chicken Z chromosome, which, like the Y, is present only in one sex (females in birds) and does not recombine with its homolog [12]. To further understand the evolution of suppressed recombination between X and Y chromosomes, we describe results from the plant genus *Silene*. This genus is a model for the study of plant sex chromosome evolution, since the sex chromosomes evolved recently [7,13].

One group of closely related dioecious *Silene* species (i.e., species with separate sexes) includes *S. latifolia, S. dioica,* and *S. diclinis,* which have an X-Y sex-determination system with a male-determining Y [1,14], while many *Silene* species are hermaphroditic or gynodioecious (i.e., some plants bear hermaphrodite flowers and others female flowers). Dioecy and sex chromosomes thus probably evolved within this genus [13]. All diploid *Silene* species have *n* = 12 chromosomes [15], so there is no evidence for neo-sex chromosome formation, although an autosomal region of unknown size has been duplicated on the Y [16].

Several sex-linked genes from S. latifolia have recently been identified and sequenced (Table 1), allowing progress in understanding the evolution of these sex chromosomes. Four genes have functional X- and Y-linked homologues. Very different X-Y divergence of two gene pairs suggested that different Y chromosome regions probably ceased recombining at different times in these species' evolutionary history [17]; testing this hypothesis requires knowing the genes' locations on the sex chromosomes. We here describe a new gene pair in *S. latifolia, SlX3* and *SlY3* (together termed locus 3; Table 1), and present the first genetic map for the X chromosomes in three dioecious species. Divergence between the X and Y chromosomal copies of the different genes indeed correlates with increased distance from the PAR, but the time scale is very different from that in mammals. Three genes (locus 3, the *SlX4-SlY4* pair [termed locus 4], and *DD44*) ceased recombining long before the three dioecious species split, whereas the X and Y copies of *SlX1-SlY1* (termed locus 1) continued to recombine until recently. We discuss the implications of these results for the mechanism of recombination arrest between the sex chromosomes.

**Table 1:**
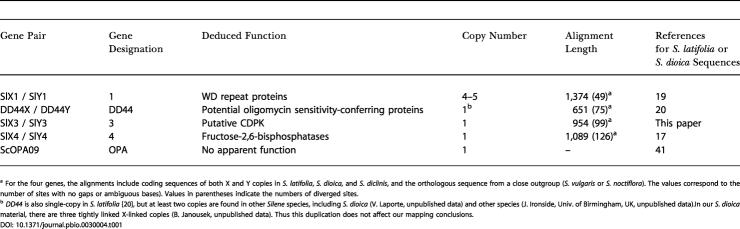
Description of the Four X-Y Gene Pairs and the PAR Marker Used in the Analyses

^a^ For the four genes, the alignments include coding sequences of both X and Y copies in S. latifolia, *S. dioica,* and S. diclinis, and the orthologous sequence from a close outgroup (S. vulgaris or S. noctiflora). The values correspond to the number of sites with no gaps or ambiguous bases). Values in parentheses indicate the numbers of diverged sites

^b^ *DD44* is also single-copy in S. latifolia [20], but at least two copies are found in other *Silene* species, including S. dioica (V. Laporte, unpublished data) and other species (J. Ironside, Univ. of Birmingham, UK, unpublished data).In our S. dioica material, there are three tightly linked X-linked copies (B. Janousek, unpublished data). Thus this duplication does not affect our mapping conclusions

## Results

### Characterization of Gene 3

Locus 3 was identified from S. latifolia cDNA. The *SlX3* open reading frame of 575 amino acids encodes a protein sequence similar to calcium-dependent protein kinases (CDPKs) from tobacco, rice, and Arabidopsis thaliana (the best BLAST hits had 75%–80% amino acid identity, based on more than three-fourths of the length). CDPKs are associated with various kinds of stress responses [18]. Thus, locus 3 is probably a sex-linked housekeeping gene, like the previously characterized X-Y-linked genes in S. latifolia [17,19].

### Phylogenetic Analysis of the Four Sex-Linked Genes

**Figure 1:**
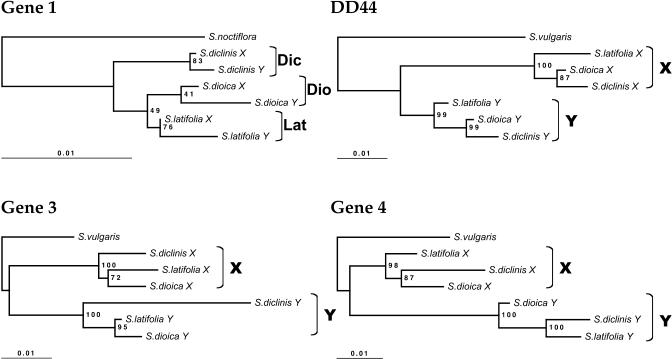
Phylogenetic Trees for *DD44* and Loci 1, 3, and 4 All trees were estimated from coding sequence alignments (using all sites except gaps) under the BIONJ method with Kimura-two-parameters corrected distances, using Phylo_Win software [43]. Other methods (maximum parsimony and ML) give very similar results. Branch lengths correspond to total sequence divergence under the model assumed (see scale bars). Values indicated at the nodes are bootstrap values exceeding 50% (based on 500 replicates). S. vulgaris was used as an outgroup (except for locus 1, for which a closer outgroup, S. noctiflora, was used). Dic = S. diclinis, Dio = S. dioica, Lat = S. latifolia. The numbers of sites analyzed are in Table 1.

Figure 1 shows the estimated phylogenetic relationships based on single X and Y copies of the four loci from each species in which sex linkage has been confirmed. Except for locus 1 (discussed below), each gene falls into distinct X and Y clades, showing that these genes ceased recombining well before the split of the present dioecious species, consistent with large X-Y divergence in both S. latifolia and S. dioica [17,20]. Not surprisingly for such closely related species [13], the phylogenies of the three dioecious species are inconsistent for these genes. For example, one Y-linked gene supports each of the possible clades *latifolia-dioica, latifolia-diclinis,* and *dioica-diclinis* (Figure 1).

Gene 1 X-Y divergence is much less than that of the other genes studied [17]. We therefore tested whether divergence between the X and Y copies started before or after the speciation event. The grouping of this gene by species in Figure 1 suggests independent *X1-Y1* divergence in the three dioecious lineages. For such closely related sequences, however, analysis using single X and Y sequences from each species confounds fixed differences between species with within-species polymorphisms, and can be misleading, given that S. latifolia is a highly variable species [21]. Ancestral polymorphisms persisting through the speciation event also obscure close phylogenetic relationships, particularly inferences using X-linked genes, which have large within-species polymorphism [7,22]. Finally, the well-documented introgression between S. latifolia and S. dioica [23] may contribute to the phylogenetic discrepancies.

**Figure 2:**
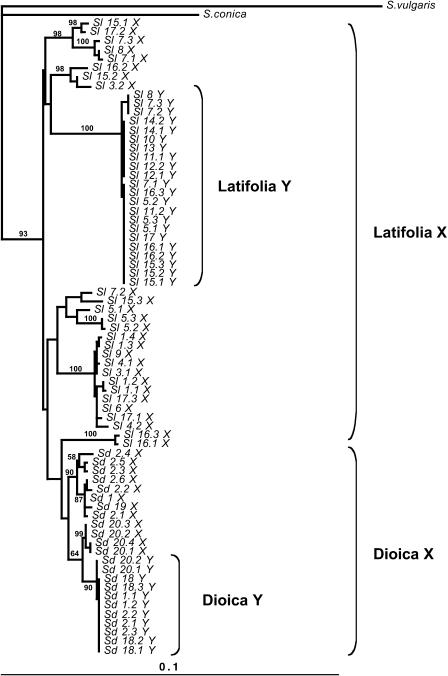
Phylogenetic Tree for Gene 1, including Within-Species Diversity The tree was estimated using PHYML software [52] from a DNA alignment including coding sequences and introns of 12 X and 11 Y S. dioica alleles, and 26 X and 22 Y from S. latifolia [22]. There were 973 sites, excluding gap regions, among which 154 variable sites were used. The estimation used the BIONJ algorithm with global gap removal. The percentage of invariant sites, the transition-transversion ratio, and the α parameter of a γ distribution of substitution rates, were estimated by the program, and we assumed four categories of evolutionary rates, to take into account the different evolutionary dynamics of coding and intron sites. The HKY substitution model was used. Bootstrap values exceeding 50% (based on 100 replicates) are indicated at the nodes, but some bootstrap values exceeding 50% for terminal nodes are omitted because of lack of space.

We therefore analyzed the *X1-Y1* gene pair separately, using multiple sequences from two species. If *X1-Y1* divergence started sufficiently long before the species split, some sites should share the same fixed differences between X and Y sequences in both S. latifolia and S. dioica. The number of such sites depends on the amount of time after recombination ceased; for the genes other than gene 1, this number is large (see above), but for gene 1 no such sites were found. If, on the other hand, *X1* and *Y1* diverged after the species split, some sites should differ between the species, but not between X and Y of the same species. This is found for mammalian and bird sex chromosomes, and phylogenetic analysis suggests that some X and Y (or, in birds, Z and W) genes ceased recombining independently in different taxa [24,25]. However, because the dioecious *Silene* species are very closely related [13], there are few fixed differences, and, using global gap removal to be conservative, none between the *X1* sequences. However, some Y variants are exclusive to each species; we found five nucleotide variants fixed only in the S. latifolia Y (plus nine indel variants), and ten fixed only in S. dioica Y (plus one indel). Since only 11 S. dioica Y sequences were analyzed, the number of fixed Y variants is probably overestimated, however (some may actually be polymorphic in this species). Furthermore, in a tree estimated excluding these sites with fixed differences in the Y-linked sequences (as is appropriate for such closely related species), the Y sequences are nested within those of the X of each species (Figure 2), implying suppression of X-Y recombination within these species. This suggests the possibility of independent cessation of recombination after speciation. However, we cannot exclude the possibility that recombination stopped shortly before the dioecious species split. Under this alternative, if the *Y1* genes retained some polymorphism, variants in the *Y1* genes would become fixed differences when Y chromosome diversity was lost within each species; according to this hypothesis, however, each species must, by chance, have retained *Y1* variants closest to its own X sequences.

### Correlation between X-Y Divergence and Position on the X Chromosome

**Figure 3:**
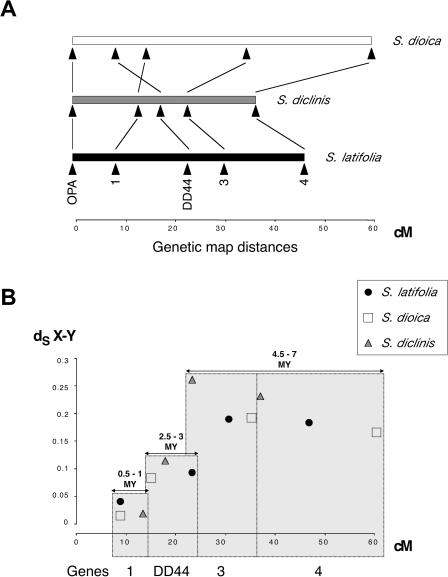
X Genetic Maps in the Three Dioecious Species versus Plot of Synonymous Divergence (A) Gene orders and the map positions of the genes. The PAR is not drawn to scale, as there is only one marker, and the map shows only the portion of the X chromosome containing our marker genes. Vertical lines connect the homologous genes in the three species, and the chromosomal rearrangement in S. dioica, are shown by crossed lines for locus 1 and *DD44*. (B) Plot of synonymous divergence between X and Y pairs (*dS*_X-Y_), estimated using PAML, against the map position using the gene order in S. latifolia and S. diclinis (see text). Synonymous divergences are statistically significantly distinguished for the following three groups of genes: locus 1, the *DD44* gene, and loci 3 and 4 (see text). The figure also indicates minimum and maximum X-Y divergence time estimates for the genes, assuming a molecular clock and 1.8 × 10^–8^ synonymous substitutions per synonymous site per year (the mean of the values 1.4 × 10^–8^ and 2.2 × 10^–8^ discussed in the text).

The gene order is the same in S. latifolia and in the S. diclinis × S. latifolia hybrid (Figure 3A). Locus 1 is closest to the PAR. If the S. diclinis and S. latifolia maps differed by an inversion or other rearrangement, the map using hybrid parents should contain a non-recombining region; this was not observed. Thus, the gene order determined in the *S. diclinis x S. latifolia* hybrid must also apply in S. diclinis. In S. dioica, however, the map order of locus 1 and *DD44* is reversed relative to the other species (Figure 3A).

Synonymous divergence *(dS)* between the X and Y sequences of S. latifolia and S. diclinis (*dS*_X-Y_) correlates with the gene's distance from the PAR in the X chromosome genetic map (Figure 3B). X-Y synonymous divergence in S. latifolia does not differ significantly between genes 3 and 4, but these genes' synonymous divergence values differ significantly from that for genes 1 or *DD44* (with *p* < 0.01). X-Y synonymous divergence also differs significantly between genes 1 and *DD44* (*p* = 0.01). These results suggest progressive suppression of the recombination between X- and Y-linked alleles of different genes. In *S. dioica,* the same correlation exists, using the S. latifolia or S. diclinis gene order; thus, the rearrangement probably arose recently in *S. dioica,* consistent with its absence in the other dioecious species. A recent rearrangement, such as an inversion, after the *DD44*-X and -Y sequences had diverged for some time, would not affect this gene's X-Y divergence relative to that of gene 1. In mouse species, where rearrangements have occurred, evolutionary strata corresponding to those on other mammalian X chromosomes are still plainly discernible [26].

### Comparing Sequence Divergence of X and Y Copies

Analysis of the coding sequences shows that all four Y-linked genes appear to encode functional sequences; in each case, the nonsynonymous divergence *(dN)* was less than *dS* for divergence between X and Y sequences (*dN*/*dS* values in Table 2); although *dN* is high for the *DD44* gene pair, it is considerably below *dS*. These results are consistent with cDNA representation of all sequences except the Y-linked copy of gene 3; despite repeated attempts, this copy never amplified from leaf cDNA, whereas the X chromosome copy amplified consistently (see Materials and Methods).

**Table 2:**
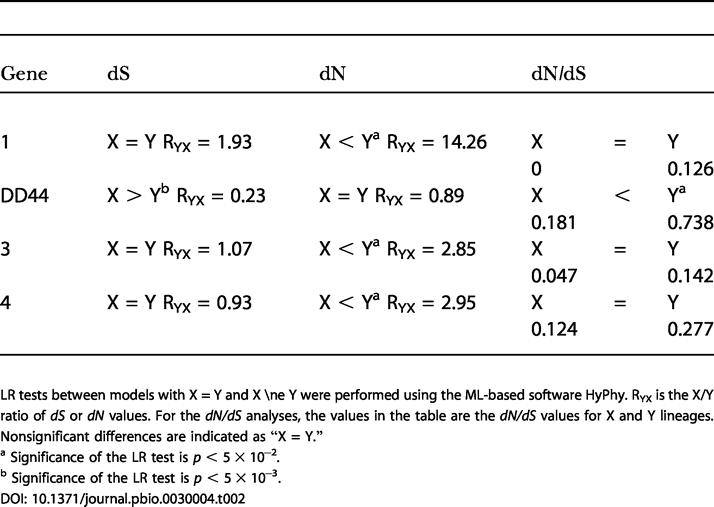
Comparison of Evolutionary Rates in the X and Y Clades

LR tests between models with X = Y and X ≠ Y were performed using the ML-based software HyPhy. R_YX_ is the X/Y ratio of *dS* or *dN* values. For the *dN/dS* analyses, the values in the table are the *dN/dS* values for X and Y lineages

Nonsignificant differences are indicated as “X = Y.”

^a^ Significance of the LR test is *p* < 5 × 10^−2^

^b^ Significance of the LR test is *p* < 5 × 10^−3^

The Y copies of all genes have higher *dS, dN,* and *dN*/*dS* values than the X-linked copies, except for *DD44* (Table 2). However, the differences are significant only for *dN*. The differences in the numbers of synonymous differences are also nonsignificant, taking into account diversity within species. Synonymous site evolution is significantly faster in *DD44*-X than in *DD44*-Y, in contrast to the other genes, where the Y tends to evolve faster than X copies (although the differences are nonsignificant; Table 2). Exon 1 of *DD44* is particularly divergent [20], but our results for this gene are similar if we exclude this exon (unpublished data). The results for gene 1 presented in Table 2 cannot be interpreted reliably because of polymorphisms within the species (see above), which would cause overestimation of numbers of substitutions. Overall, therefore, *dN* is clearly higher in the Y copies of genes 3 and 4, but its mutation rate is not higher, since X-Y differences in *dS* are nonsignificant; combining the probabilities from the likelihood ratio (LR) tests for these two genes, the *dN*/*dS* difference between Y and X is highly significant (χ^2^ = 11.7, with 4 degrees of freedom). Our observation of similar *dS* values contrasts with previous analyses [27], probably because we used only synonymous sites, rather than synonymous plus noncoding sites. The *S. diclinis Y3* gene also seems to evolve faster than the other *Y3* genes (see Figure 1); for this gene, the difference is seen for both synonymous sites (6-fold increase) and nonsynonymous ones (3.6-fold increase), but it is significant only for synonymous sites.

## Discussion

### Progressive Differentiation of the X and Y Chromosomes

The correlation of *dS*_X-Y_ of these dioecious plants with distances from the PAR in the X chromosome genetic map suggests that suppression of recombination between X and Y genes progressed, starting from an “ancient” sex chromosomal region (presumably containing the primary sex determining loci) and moving toward the current PAR. This pattern resembles the “evolutionary strata” for mammalian X-Y gene pairs based on *K*_s_ values, a measure of divergence per site similar to *dS* [9,10]. However, the time scale is much shorter for the plant sex chromosomes. The largest *dS*_X-Y_ values among our four gene pairs is 26% for locus 3 in S. diclinis. This overlaps the values for the mammalian stratum 4 and 3 genes (mean *K*_s_ values 8% and 30%, respectively); these strata are inferred to have ceased recombining between the X and Y 30–50 million years ago (MYA) for stratum 4, and 80–130 MYA for stratum 3, whereas strata 1 and 2 diverged 130–320 MYA [9,11].

The *S. latifolia, S. dioica,* and S. diclinis X-Y sequence divergence data show that X-Y differentiation was already advanced in the common ancestor of these species, except for locus 1. The maximum synonymous X-Y divergence observed for our genes is approximately 25%, including *SlAp3,* which probably transposed from an autosome onto the Y soon after the sex chromosomes evolved [16]; all these genes appear to be functional. This divergence is also similar to that for *MROS3*-X/Y, whose Y-linked copy is degenerated [6]. Unless genes with higher divergence are discovered in the male-determining region of Y chromosomes of dioecious *Silene* species, the *Silene* sex chromosomes must have evolved much more recently than mammalian sex chromosomes.

There are few reliable absolute molecular clock calibrations in plants [28], and none for *Silene*. For the nuclear genes *Chs* and *Adh* in the family Brassicaceae, estimated rates are, respectively, 1.4 × 10^–8^ to 2.2 × 10^–8^ substitutions per synonymous site per year [29], and a similar value was estimated for *Ipomoea* [30]. Using synonymous site divergence values suggests an age estimate of 5–10 MYA for the sex chromosomes of the S. latifolia group of species. However, substitution rates for some plant *Adh* genes are almost ten times slower, particularly for plants with long generation times [31]. Thus, a greater age cannot be excluded. It is nevertheless clear that the X and Y copies of genes3, 4, and *DD44* differentiated before the *S. dioica–S. latifolia–S. diclinis* speciation, whereas gene 1 may have ceased recombining after this, perhaps independently in S. latifolia and *S. dioica;* no analysis can be done in S. diclinis without diversity data for this species, but suppression of X-Y recombination within this species after its split from the other dioecious species is also possible (Figure 1). If this event occurred shortly before the dioecious species split, our results show that it must have happened in such a way that the Y-linked copy of gene 1 retained some diversity, in other words, by some mechanism other than an inversion (see below). Suppression of X-Y recombination (diminution of the PAR) has occurred quickly, and probably independently, in different mammalian and bird lineages [24,25,32].

The mechanism suppressing X-Y recombination is unknown. Recombination could be reduced either by inversions (or other major recombination rate changes), and/or by modifiers reducing local crossover rates. The “strata” of different divergence in mammalian sex chromosomes may have resulted from a series of Y inversions disrupting X-Y recombination [9]. Inversions exist between human X and Y chromosomes [10], but have not yet been explicitly related to the strata, so they may not be the sole cause of the divergence differences. Moreover, new pairs of X-Y linked genes recently analyzed do not suggest clear-cut boundaries between strata; divergence values for strata 3 and 4 genes are not discontinuous [10]. Finally, the *amelogenin* gene, at the strata 3–4 boundary, is not disrupted by an inversion [25]. Thus, gradual modification of recombination rates may have played a part in reducing recombination in some regions of the X-Y pair, in both *Silene* and mammals. Testing this for the dioecious *Silene* species requires a Y-chromosome map. The present map, based on deletion mutants in *Silene* [20,33], requires further markers and deletions for detailed comparison with the genetic map of the X chromosome. S. latifolia Y deletion mutants with altered meiotic X-Y pairing (unpublished data) suggest that the S. latifolia Y may carry genes suppressing recombination, and should help test whether mechanisms other than inversions contributed to reduction of the PAR.

The mechanism of recombination reduction between X and Y chromosomes is important for understanding the diversity in loci that recently ceased recombining, such as gene 1 in *Silene*. Recombination suppression may be selectively favored to preserve advantageous Y-linked combinations of alleles at different loci, such as genes that are advantageous in males but not in females [34], although it seems unlikely that all three dioecious species studied here could recently have acquired advantageous Y-linked genes. Involvement of selectively favored inversions causing the formerly pseudoautosomal gene 1 to become Y-linked might be detectable from sequence data, since a selective sweep would be expected. This would contribute to low diversity for all the Y-linked genes, consistent with the long branches in Y lineages (Figures 1 and 2). However, although Y-linked diversity is low, there is no evidence of such events in the frequency spectra of the genes [7,22].

### Degeneration of the Y Chromosome

Our analyses suggest that both reduction of recombination and Y degeneration may be in progress for *Silene* sex chromosomes. Degeneration is likely, since genotypes with a Y but no X chromosome are inviable [1,35], but so far, only one degenerated plant Y-linked gene has been found, *MROS3*-Y in S. latifolia [6]. The extent of genetic degeneration and gene loss in the *Silene* Y is uncertain, because most currently known sex-linked genes in these plants were ascertained from a cDNA-based search for Y-linked genes. Bacterial artificial chromosome clone sequencing may provide unbiased comparisons of homologous X- and Y-linked regions, and this has been started in papaya [4]. Some degeneration of Y-linked genes in *Silene* can also be inferred when *dN* values in the Y are elevated compared with X lineages. This is seen for the two “old” *Silene* Y-linked genes, locus 3 and locus 4 (Table 2). Differences in *dS* are systematically lower than in *dN* (the ratio of *dS* values for X and Y lineages is close to 1, but *dN* is roughly 3-fold larger overall for Y lineages). Thus the higher *dN* in the Y-linked alleles is not due to a higher mutation rate (higher *dS*) in the Y than the X. Moreover, the Y-linked copy of locus 3 fails to amplify in RT-PCR experiments, and may be degenerated.

These observations, plus those for gene 1 (see Results), suggest that Y copies of genes loci 1, 3, and 4 evolve faster than X copies, due either to a higher rate of fixation of advantageous mutations in the Y copies or to accumulation of slightly deleterious amino acid variants in the Y copies (Y degeneration). To discriminate between these hypotheses, McDonald-Kreitman tests can be done to compare fixed differences (divergence) and polymorphisms and test for an excess of selectively advantageous nonsynonymous substitutions [36]. At present, this is possible only for genes 1 and 4; there were no nonsynonymous polymorphisms for *DD44,* and no diversity data have yet been obtained for gene 3. The result of this test was nonsignificant; there is thus no evidence that *Y1* and *Y4* evolution is driven by selection. There is, however, very low polymorphism in the Y copies, so the test has low power [27].

Genetic degeneration is supported by low levels of polymorphism of Y- compared with X-linked genes, taking into account the lower Y effective population size [7,22]. This difference is predicted in a degenerating Y chromosome, because various hitchhiking processes leading to degeneration, including selective sweeps, background selection, and weak Hill-Robertson effects [5] reduce diversity, even at loci that are not themselves degenerating.

Why is degeneration so slight for our *Silene* Y-linked genes? Our analyses suggest that degeneration of the genes studied here is partial, at most, consistent with a recent origin of the *Silene* sex chromosomes. However, there has probably been enough time for degeneration, since this occurred rapidly for genes on the neo-Y chromosomes of D. miranda [8], which are much younger than the *Silene* Y. *Silene* sex chromosomes are more advanced in sex chromosome evolution than in some other plants. The papaya sex-determining region is just a small nonrecombining part of one chromosome, yet there is evidence for considerable differentiation, including addition of repeat sequences and some evidence for gene loss [4]. More likely, the Y-linked genes we have studied (which are all housekeeping genes) are under selective constraints. The lower effective population size of Y-linked genes, and thus the expected reduced efficacy of selection ([5]) may thus be too slight to allow the Y copies of these genes to lose function, but merely allows higher amino acid substitution. Our findings parallel those for most loci on the D. miranda neo-Y chromosome [37], the bird W chromosome [38], and in other situations in which effective population size in reduced, such as protein-coding genes of the endosymbiont *Buchnera* [39]. In all these cases, genes evolving without recombination retain homology with their ancestral copies, but undergo faster amino acid replacement (including several frameshift and deletion mutations in the D. miranda neo-Y [37]), suggesting that the common factor is weakened ability of natural selection to preserve adaptation.

## Materials and Methods

### 

#### Plants used and nucleic acid extraction.

S. latifolia plants were from Edinburgh (D. Charlesworth personal collection) and from Fontainebleau forest (France). S. dioica plants were collected in Corrèze (France). S. dioica plants from the *Sherringham* population (Sherringham, England), used for isolation of the *ScOpa09* marker, were kindly provided by D.L. Mulcahy (Department of Biology, University of Massachussetts). S. noctiflora and S. vulgaris were obtained from the seed collection of the Lyon Botanical Garden (Lyon, France). Seeds of S. diclinis were obtained from the seed collection of the Institute of Biophysics in Brno (Czech Republic). Interspecific hybrid Silene diclinis × *latifolia* plants were generated by pollination of a S. diclinis female with pollen of an MAV line male (*S. latifolia*) kindly provided by S. Matsunaga (Department of Biotechnology, Osaka University). The S. latifolia U9 line, which was used for pollination of the interspecific hybrid, was kindly provided by S. Grant (Department of Biology, University of North Carolina).

Genomic DNA was extracted from leaves as described [19]. For RT-PCR from total leaf RNA, first-strand cDNA was reverse transcribed using RevertAid M-MuLV RT (Fermentas, Vinius, Lithuania) and the oligo-dT primer T11VN (5′- TTTTTTTTTTTVN-3′).

#### Isolation of *SlX3*/*SlY3*

Locus 3 was identified in S. latifolia by the approach that yielded loci 1 (*SlX1*/*SlY1* [19]) and 4 (*SlX4*/*SlY4* [17]). From an initial partial cDNA sequence of a clone that hybridized to a probe containing Y-linked sequences, both 3′ and 5′ RACE-PCR were performed [19], and the final coding sequence was obtained by sequencing the RT-PCR product obtained using primers 11S10 (5′- ATCACCATCATCATTTCCACC-3′) and 11AS11 (5′- CAGTGAAATCTTTCATTTACCACG-3′). Segregation analysis (see below) showed that this sequence corresponds to *SlX3*. The *SlY3* sequence was obtained from genomic DNA by PCR genomic walking [40], using the specific primers 11AS15 (5′- TCAGTGTCTCCTTGAGTTTCTTGCAC-3′) and 11AS15C (5′- TGCACAAGATGGACTGGCTACAATACG-3′) for the first and second PCR, respectively, and Ex Taq polymerase (Takara Bio, Otsu, Shiga, Japan) for both PCR reactions. Similarly to gene 4 [17], Southern blot analysis showed that gene 3 is present as a single copy in the S. latifolia genome.

#### Amplification and sequencing of orthologous sequences.

The orthologs of each of the four gene pairs in Table 1 were amplified in *S. dioica, S. diclinis,* and S. noctiflora or S. vulgaris using primers designed from S. latifolia sequences (Table 3, which also provides GenBank accession numbers). All sequences were amplified from cDNA, except for *Y3,* which was amplified from genomic DNA. The PCR conditions, using Taq polymerase (Amersham Pharmacia, Piscataway, New Jersey, United States), were as follows. One incubation at 94 °C for 5 min; 35 cycles of: denaturation at 94 °C for 30 s, annealing at a temperature that depended on the primers for 30 s, and elongation at 72 °C for a time depending on the length of the amplicon (Table 3); and a final extension at 72 °C for 5 min.

**Table 3:**
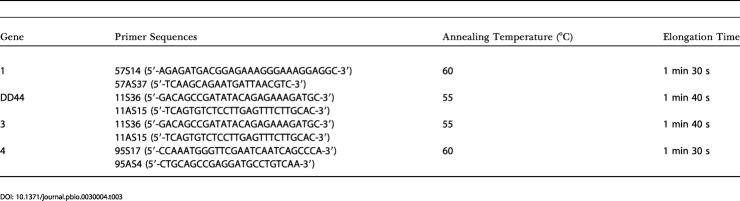
Primers and Annealing Temperatures Used for RT-PCR Amplification of the Genes Listed in Table 1

All genes, except gene 1, are single-copy (see Table 1). In *S. latifolia,* three or four copies of gene 1 are detected in Southern blots [19], and the A. thaliana genome has five copies, *AtMSI4* (GenBank accession number AF028711) being the probable ortholog of *SlY*/*X1*. In *S. latifolia,* some of the copies have been shown not to be sex-linked (F. Monèger, personal communication). Southern blots were not done in S. dioica or *S. diclinis,* but RT-PCR reactions in all the dioecious species always amplified a single sequence in the females plus another very similar sequence in males, clearly representing the expected X and Y copies. Thus, for the analyses presented later, this gene can be treated as a single-copy gene, as was also the case in previous mapping work [20].

For locus 3, the 3′ two-thirds of the coding sequences of *S. dioica, S. diclinis,* and S. vulgaris were amplified, either by RT-PCR (X copies) or from genomic DNA (Y copies)*.* PCR products were cloned into pGEM-T Easy vector (Promega, Madison, Wisconsin, United States), and multiple clones were sequenced for each gene. Sequencing reactions were carried out with ABI Big Dye Terminator V1.1 DNA sequencing kit, on an Applied Biosystems 3100 sequencer (Applied Biosystems, Foster City, California, United States).

#### Sex linkage and genetic mapping.

**Figure 4:**
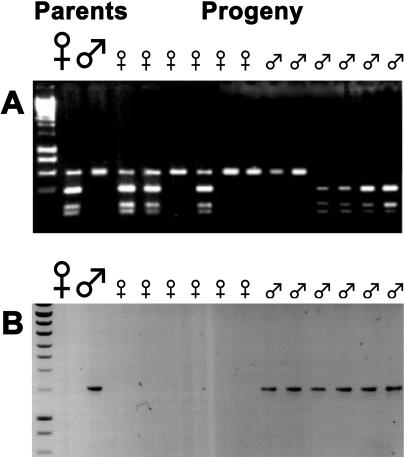
Segregation Analysis of Locus 3 in S. dioica To test for sex linkage, the female and male parents of the family and six progeny of each sex were genotyped. Genomic DNA preparations from these plants were used in PCR amplifications. The PCR products were separated by electrophoresis and visualized using ethidium bromide. (A) With primers specific for the *X3* allele, the restriction enzyme RsaI reveals an allelic polymorphism. The maternal plant is heterozygous and has both cut and uncut alleles while the male parent has only the uncut allele. Female progeny always have the uncut allele (like the father), and male progeny have one of the maternal alleles, but never both, corresponding to the expected segregation of the X chromosome. (B) Primers specific for the Y gametolog. A product amplifes only with male DNA, corresponding to the expected segregation of the Y chromosome.

Sex linkage of three gene pairs studied here was previously demonstrated in either S. dioica or S. latifolia. We have now confirmed sex linkage of all four loci by segregation analysis in all three dioecious species (Figure 4), and, for genes 1, 4, and *DD44,* by population studies using allele-specific PCR reactions to show that the putative Y-linked alleles are consistently present only in males, while the X-linked ones amplify in both sexes [17,22] and unpublished data).

Only X-linked genes can be mapped genetically, because the Y chromosome recombines with the X only in the PAR. For each locus, gene-specific primers were used to amplify X alleles from genomic DNA of potential seed parents. The PCR product was directly sequenced and the chromatograms inspected for polymorphisms scorable by restriction enzyme digestion (Table 4). Progeny of heterozygous mapping family females were sexed and genotyped for the maternal alleles. In *S. diclinis,* no suitable polymorphisms were found, so the loci were ordered in a S. diclinis × S. latifolia hybrid plant pollinated by a S. latifolia male.

**Table 4:**
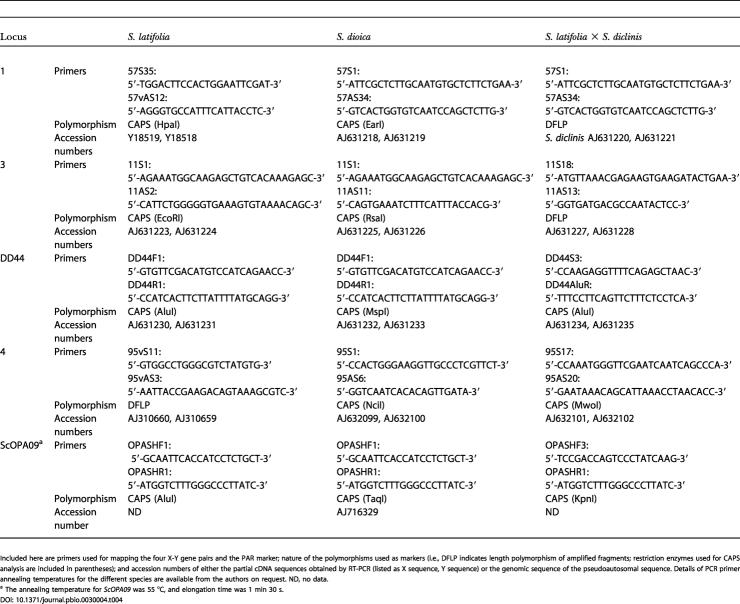
Primers Used for Mapping

Included here are primers used for mapping the four X-Y gene pairs and the PAR marker; nature of the polymorphisms used as markers (i.e., DFLP indicates length polymorphism of amplified fragments; restriction enzymes used for CAPS analysis are included in parentheses); and accession numbers of either the partial cDNA sequences obtained by RT-PCR (listed as X sequence, Y sequence) or the genomic sequence of the pseudoautosomal sequence. Details of PCR primer annealing temperatures for the different species are available from the authors on request. ND, no data

^a^ The annealing temperature for *ScOPA09* was 55 °C, and elongation time was 1 min 30 s

To orient the X genetic map, we used a pseudoautosomal marker. For this, we cloned and sequenced a RAPD fragment incompletely linked to the X chromosome of the pollen donor of the S. dioica family in which this marker was originally developed [41]. The sequence encodes a protein with similarity to an Oryza sativa putative non-long terminal repeat reverse transcriptase (E value = 2.5 × 10^–10^; accession number Q9FW98 in the UniProt/TrEMBL database, but contains stop codons. Primers (ScOPA09F1: 5′- GCAATTCACCATCCTCTGCT-3′ and ScOPA09R1: (5′- ATGGTCTTTGGGCCCTTATC-3′) were designed from this sequence. In plants grown from seeds of this family, presence or absence of the expected amplified fragment accords exactly with results using the original pseudoautosomal RAPD marker primer OPA09. With our primers, S. dioica plants from the Corrèze population were genotyped by digesting PCR products with the restriction enzyme TaqI; the recombination frequency between the marker locus and sex was approximately 2.5%, confirming the pseudoautosomal location. In *S. latifolia,* genotyping was done using the same primers and an AluI site polymorphism. Genotype data were analyzed by both three-point and multipoint mapping, using JoinMap version 1.4 [42]. Thus the gene orders are well established; Table S1 gives estimated genetic distances between markers and their standard errors.

#### Phylogenetic analysis.

The primer sequences were removed before sequence analyses. For each gene, the nucleotide sequences were aligned using the corresponding amino acid sequences as a guide, using ClustalW with the Seaview interface (http://pbil.univ-lyon1.fr/) [43]. Alignment lengths are given in Table 1.

Phylogenetic trees were estimated including all sites except those with gaps by neighbor joining (NJ), maximum parsimony, and maximum likelihood (ML), using Phylo_Win (http://pbil.univ-lyon1.fr/) [43]. For NJ trees, we used Kimura two-parameter corrected distances; results with other distances corrected for multiple hits were similar, as the sequences are not highly diverged and have similar GC content (unpublished data). Branches were tested by bootstrapping (500 replicates). Trees were edited with NJplot (http://pbil.univ-lyon1.fr/) [44] and TreeView (http://taxonomy.zoology.gla.ac.uk/rod/treeview.html) [45].

#### Divergence analysis.

Both *dS* and *dN* site divergences were estimated using PAML 3.13 (http://abacus.gene.ucl.ac.uk/software/paml.html) [46] and JaDis (http://pbil.univ-lyon1.fr/) [47]. Estimates of *dS* and *dN* are similar under various substitution models (namely, Goldman and Yang 1994 [46], Yang and Neilson 2000 [48], and Nei and Gojobori 1986 [49], implemented in PAML; and Li 1993 [50] using JaDis). We report values from the ML approach based on the Goldman and Yang 1994 codon-based model [46].

Values for *dS* or *dN* of X and Y sequences were compared using HyPhy 0.99 (Kosakovsky Pond, personal communication; http://www.hyphy.org, using the alignment and NJ tree for each gene, including the outgroup species (Figure 4) to polarize the synonymous and nonsynonymous substitutions between X and Y genes into X-specific and Y-specific lineages, using ML. To build the likelihood function, we used the MG94xHKY85 codon-based substitution model with different transversion and transition rates. We compared *dS* values under two models for each gene. Model 1 (“relative synonymous rates”) expresses *dS* values for Y lineages as multiples of values for X lineages: R_syn_ = *dS*_Y_/*dS*_X_. In model 2 (“equal synonymous rates”), R_syn_ was constrained to be equal to 1 (*dS*_Y_ = *dS*_X_). We compared the ML values by a LR test with model 2 as the null hypothesis. We used the same approach to compare *dN* values (with *dN/dS* replacing R_syn_). To compare *dN/dS* using LR tests, we again defined two models. Model 1 assumed two global variables (*dN*/*dS*)_X_ and (*dN*/*dS*)_Y_ so that the nonsynonymous rates of branches of the X lineage were expressed in terms of the synonymous rate (*dN*/*dS*)_X_, and similarly using (*dN*/*dS*)_Y_ for Y branches (“shared *dN*/*dS*”), while model 2 (“shared and equal *dN*/*dS*”) assumed (*dN*/*dS*)_X_ = (*dN*/*dS*)_Y_.

To test whether *S. diclinis Y3* evolves faster than other *Y3* sequences, we assumed a common R_syn_ for S. dioica and *S. latifolia,* as in model 1 above, but added a further parameter, the *dS*_Y_/*dS*_X_ ratio for S. diclinis (model 1*). We compared models 1 and 1* using a LR test as above; we tested *dN* and *dN*/*dS* differences similarly.

McDonald-Kreitman tests were done using DNAsp software, version 3.95 [51]. The divergence and polymorphism data used are from previous work and were available only for genes 1 and 4 [17]; there were no nonsynonymous polymorphisms for *DD44,* and no diversity data are yet available for gene 3.

To test for differences in divergence between the X and Y sequences of different genes, we compared numbers of fixed X-Y differences in S. latifolia by contingency tests, using DNAsp. To infer fixed differences rigorously, we used diversity data within S. latifolia for genes 1, 4, and *DD44*. For gene 3 no diversity data are yet available; however, because this gene pair has high X-Y divergence, raw divergence values should suffice, so for this gene we estimated numbers of differences from single X and Y sequences.

#### Analysis of gene 1.

To test whether X and Y sequences of gene 1 continued recombining and started to diverge after the dioecious species split, a C program was written to find fixed differences in a set of multiple S. latifolia and S. dioica Y and X sequences, plus one sequence from each of two outgroup species, S. vulgaris and *S. conica;* this enables us to identify whether the changes were in the X or Y lineages, using parsimony. With global gap-removal, the program unambiguously distinguishes fixed differences, including insertions and deletions, from polymorphisms within species. The outgroup sequences are shorter than the other sequences, so some fixed differences in the S. dioica Y could not be analyzed.

As this dataset includes the first approximately 2,000 sites of gene 1, including coding and intron sequences [22], a more sophisticated model for sequence evolution is required for phylogenetic analysis than for the coding sequences analyzed above. We estimated the percentage of invariant sites, and the transition to transversion ratio, and fitted a GAMMA distribution, estimating its ALPHA parameter with four categories of sites evolving at different rates, using the HKY (Hasegawa, Kishino, Yano [52]) model as the global substitution model. The tree was estimated using NJ (BIONJ) with global gap removal, using a fast ML-based program, PHYML (http://www.lirmm.fr/guindon/phyml.html) [53], excluding fixed differences from the multiple alignment (to avoid conflicting phylogenetic signals between fixed and polymorphic differences). The statistical support for the tree was estimated by bootstrapping (100 replicates), using SEQBOOT, followed by CONSENSE to make a consensus tree with the resulting 100 PHYML trees.

## Supporting Information

Table S1 Recombination Fractions (Rf) between the Loci, and Standard Errors of Rf Values (36 KB DOC).

### Accession Numbers

The GenBank (http://www.ncbi.nlm.nih.gov/) accession number for *AtMSI4* is AF028711; the UniProt/TrEMBL (http://www.ebi.ac.uk/trembl/) accession number the *ScOpa09* marker is Q9FW98.

## Abbreviations

CDPK: calcium-dependent protein kinase
cM: centimorgans
*dN*: nonsynonymous divergence per site
*dS*: synonymous divergence per site
*dS*_X_: *dS* for the X lineage
*dS*_X-Y_: *dS* between the X and Y sequences
*dS*_Y_: *dS* for the Y lineage
LR: likelihood ratio
ML: maximum likelihood
MYA: million years ago
NJ: neighbor joining
PAR: pseudoautosomal region
RAPD: rapid amplification of polymorphic DNA
R_syn_: *dS*_Y_/*dS*_X_

## References

[pbio-0030004-b01] Westergaard M (1958). The mechanism of sex determination in dioecious plants. Adv Genet.

[pbio-0030004-b02] Volff JN, Schartl M (2001). Variability of genetic sex determination in poeciliid fishes. Genetica.

[pbio-0030004-b03] Charlesworth B, Charlesworth D (1978). A model for the evolution of dioecy and gynodioecy. Amer Nat.

[pbio-0030004-b04] Liu Z, Moore PH, Ma H, Ackerman CM, Ragiba M (2004). A primitive Y chromosome in Papaya marks the beginning of sex chromosome evolution. Nature.

[pbio-0030004-b05] Charlesworth B, Charlesworth D (2000). The degeneration of Y chromosomes. Philos Trans R Soc Lond B Biol Sci.

[pbio-0030004-b06] Guttman DS, Charlesworth D (1998). An X-linked gene has a degenerate Y-linked homologue in the dioecious plant Silene latifolia. Nature.

[pbio-0030004-b07] Filatov DA, Monéger F, Negrutiu I, Charlesworth D (2000). Evolution of a plant Y-chromosome: Variability in a Y-linked gene of Silene latifolia. Nature.

[pbio-0030004-b08] Bachtrog D (2003). Adaptation shapes patterns of genome evolution on sexual and asexual chromosomes in *Drosophila*. Nat Genet.

[pbio-0030004-b09] Lahn BT, Page DC (1999). Four evolutionary strata on the human X chromosome. Science.

[pbio-0030004-b10] Skaletsky H, Kuroda-Kawaguchi T, Minx PJ, Cordum HS, Hillier L (2003). The male-specific region of the human Y chromosome is a mosaic of discrete sequence classes. Nature.

[pbio-0030004-b11] Waters PD, Duffy B, Frost CJ, Delbridge ML, Graves JAM (2001). The human Y chromosome derives largely from a single autosomal region added to the sex chromosomes 80–130 million years ago. Cytogenet Cell Genet.

[pbio-0030004-b12] Lawson-Handley LJ, Ceplitis H, Ellegren H (2004). Evolutionary strata on the chicken z chromosome: Implications for sex chromosome evolution. Genetics.

[pbio-0030004-b13] Desfeux C, Maurice S, Henry JP, Lejeune B, Gouyon PH (1996). Evolution of reproductive systems in the genus Silene. Proc R Soc Lond B Biol Sci.

[pbio-0030004-b14] Lebel-Hardenack S, Grant S (1997). Genetics of sex determination in flowering plants. Trends Plant Sci.

[pbio-0030004-b15] Goldblatt P (1981). Index to Plant Chromosome Numbers 1975–1978. St.

[pbio-0030004-b16] Matsunaga S, Isono E, Kejnovsky E, Vyskot B, Kawano S (2003). Duplicative transfer of a MADS box gene to a plant Y chromosome. Mol Biol Evol.

[pbio-0030004-b17] Atanassov I, Delichère C, Filatov DA, Charlesworth D, Negrutiu I (2001). A putative monofunctional fructose-2,6-bisphosphatase gene has functional copies located on the X and Y sex chromosomes in white campion *(Silene latifolia)*. Mol Biol Evol.

[pbio-0030004-b18] Hrabak EM, Chan CWM, Gribskov M, Harper JF, Choi JH (2003). The *Arabidopsis* CDPK-SnRK superfamily of protein kinases. Plant Physiol.

[pbio-0030004-b19] Delichère C, Veuskens J, Hernould M, Barbacar N, Mouras A (1999). SlY1, the first active gene cloned from a plant Y chromosome, encodes a WD-repeat protein. EMBO J.

[pbio-0030004-b20] Moore RC, Kozyreva O, Lebel-Hardenack S, Siroky J, Hobza R (2003). Genetic and functional analysis of DD44, a sex-linked gene from the dioecious plant *Silene latifolia* provides clues to early events in sex chromosome evolution. Genetics.

[pbio-0030004-b21] Vellekoop P, Buntjer JB, Maas JW, van Brederode J (1996). Can the spread of agriculture in Europe be followed by tracing the spread of the weed Silene latifolia. A RAPD study. Theor Appl Genet.

[pbio-0030004-b22] Filatov DA, Laporte V, Vitte C, Charlesworth D (2001). DNA diversity in sex linked and autosomal genes of the plant species Silene latifolia and S. dioica. Mol Biol Evol.

[pbio-0030004-b23] Goulson D, Jerrim K (1997). Maintenance of the species boundary between Silene dioica and S. latifolia (red and white campion). Oikos.

[pbio-0030004-b24] Ellegren H, Carmichael A (2001). Multiple and independent cessation of recombination between avian sex chromosomes. Genetics.

[pbio-0030004-b25] Iwase M, Satta Y, Hirai Y, Hirai H, Imai H (2003). The amelogenin loci span an ancient pseudoautosomal boundary in diverse mammalian species. Proc Natl Acad Sci U S A.

[pbio-0030004-b26] Sandstedt SA, Tucker PK (2004). Evolutionary strata on the mouse X chromosome correspond to strata on the human X chromosome. Genome Res.

[pbio-0030004-b27] Filatov DA, Charlesworth D (2002). Substitution rates in the X- and Y-linked genes of the plants, Silene latifolia and S. dioica. Mol Biol Evol.

[pbio-0030004-b28] Gaut BS (1998). Molecular clocks and nucleotide substitution rates in higher plants. Evol Biol.

[pbio-0030004-b29] Koch M, Haubold B, Mitchell-Olds T (2000). Comparative evolutionary analysis of chalcone synthase and alcohol dehydrogenase loci in *Arabidopsis, Arabis* and related genera (Brassicaceae). Mol Biol Evol.

[pbio-0030004-b30] Durbin ML, Learn GH, Huttley GA, Clegg MT (1995). Evolution of the chalcone synthase gene family in the genus *Ipomoea*. Proc Natl Acad Sci U S A.

[pbio-0030004-b31] Gaut BS, Morton BR, McCaig BC, Clegg MT (1996). Substitution rate comparisons between grasses and palms: Synonymous rate differences at the nuclear gene *Adh* parellel rate differences at the plastid gene *rbcL*. Proc Nat Acad Sci U S A.

[pbio-0030004-b32] Marais G, Galtier N (2003). Sex chromosomes: How X-Y recombination stops. Curr Biol.

[pbio-0030004-b33] Lebel-Hardenack S, Hauser E, Law TF, Schmid J, Grant S (2002). Mapping of sex determination loci on the white campion *(Silene latifolia)* Y chromosome using amplified fragment length polymorphism. Genetics.

[pbio-0030004-b34] Rice WR (1997). The accumulation of sexually antagonistic genes as a selective agent promoting the evolution of reduced recombination between primitive sex-chromosomes. Evolution.

[pbio-0030004-b35] Ye D, Installé P, Ciuperescu C, Veuskens J, Wu Y (1990). Sex determination in the dioecious *Melandrium* . I. First lessons from androgenic haploids. Sex Plant Repr.

[pbio-0030004-b36] McDonald JH, Kreitman M (1991). Accelerated protein evolution at the *Adh* locus in *Drosophila*. Nature.

[pbio-0030004-b37] Bachtrog D, Charlesworth B (2002). Reduced adaptation of a non-recombining neo-Y chromosome. Nature.

[pbio-0030004-b38] Fridolfsson A-K, Ellegren H (2000). Molecular evolution of the avian *CHD1* genes on the Z and W sex chromosomes. Genetics.

[pbio-0030004-b39] Herbeck JT, Funk DJ, Degnan PH, Wernergreen JJ (2003). A conservative test of genetic drift in the endosymbiotic bacterium *Buchnera* Slightly deleterious mutations in the chaperonin groEL. Genetics.

[pbio-0030004-b40] Devic M, Albert S, Delseny M, Roscoe TJ (1997). Efficient PCR walking on plant genomic DNA. Plant Physiol Biochem.

[pbio-0030004-b41] DiStilio VS, Kesseli R, Mulcahy DL (1998). A pseudoautosomal random amplified polymorphic DNA marker for the sex chromosomes of Silene dioica. Genetics.

[pbio-0030004-b42] Stam P (1993). Construction of integrated genetic-linkage maps by means of a new computer package - JoinMap. Plant J.

[pbio-0030004-b43] Galtier N, Gouy M, Gautier C (1996). PHYLO_WIN: Two graphic tools for sequence alignment and molecular phylogeny. Comput Appl Biosci.

[pbio-0030004-b44] Perrière G, Gouy M (1996). WWW-query: An on-line retrieval system for biological sequence banks. Biochimie.

[pbio-0030004-b45] Page RDM (1996). TreeView: An application to display phylogenetic trees on personal computers. Comput Appl Biosci.

[pbio-0030004-b46] Goldman N, Yang Z (1994). A codon-based model of nucleotide substitution form protein-coding DNA sequences. Mol Biol Evol.

[pbio-0030004-b47] Goncalves I, Robinson M, Perriere G, Mouchiroud D (1999). JaDis: Computing distances between nucleic acid sequences. Bioinformatics.

[pbio-0030004-b48] Yang ZH, Neilson R (2000). Estimating synonymous and nonsynonymous substitution rates under realistic evolutionary models. Mol Biol Evol.

[pbio-0030004-b49] Nei M, Gojobori T (1986). Simple methods for estimating the numbers of synonymous and nonsynonymous nucleotide substitutions. Mol Biol Evol.

[pbio-0030004-b50] Li W-H (1993). Unbiased estimation of the rates of synonymous and nonsynonymous substitution. J Mol Evol.

[pbio-0030004-b51] Rozas J, Rozas R (1999). DnaSP version 3.0: An integrated program for molecular population genetics and molecular evolution analysis. Bioinformatics.

[pbio-0030004-b52] Yano, Hasegawa M, Kishino H, Yano TA (1985). Dating of the human ape splitting by a molecular clock of mitochondrial-DNA. J Mol Evol.

[pbio-0030004-b53] Guindon S, Gascuel O (2003). A simple, fast, and accurate algorithm to estimate large phylogenies by maximum likelihood. Syst Biol.

