# Supplementary material for: A Gradual Process of Recombination Restriction in the Evolutionary History of the Sex Chromosomes in Dioecious Plants
Source: PLoS Biol. 2004 Dec 21;3(1):e4. doi: 10.1371/journal.pbio.0030004 (PMC536007; doi:10.1371/journal.pbio.0030004)
Supplement: Table S1 — (36 KB DOC). [file pbio.0030004.st001.doc]

# Nicolas et al. Supplementary Materials

**Table S1. Recombination fractions (Rf) between the loci, and standard errors of Rf values.**

|  |  |  |  |  |  |  | |  |
| --- | --- | --- | --- | --- | --- | --- | --- | --- |
|  |  |  |  |  |  |  | |  |
|  |  |  |  |  |  |  | |  |
|  | *S. latifolia* |  | *S. dioica* |  | *S. diclinis x latifolia* | | |  |
|  | Rf (%) | SE (%) | Rf (%) | SE (%) | Rf (%) | SE (%) | |  |
|  |  |  |  |  |  |  | |  |
| locus4-locus3 | 17.3 | 3.6 | 23.9 | 4.4 | 12.5 | 5.8 | |  |
| locus4-DD44* | 18.9 | 3.7 | 41.1 | 6.0 | 15.6 | 6.4 | |  |
| locus4-locus1 | 32.4 | 4.5 | 40.2 | 5.1 | 20.6 | 6.9 | |  |
| locus4-ScOpa09 | 45.2 | 7.6 | 47.0 | 5.5 | 32.4 | 8.0 | |  |
|  |  |  |  |  |  |  | |  |
| locus3-DD44* | 9.1 | 2.7 | 28.7 | 3.6 | 5.4 | 3.7 | |  |
| locus3-locus1 | 19.6 | 3.8 | 20.6 | 4.2 | 10.0 | 4.7 | |  |
| locus3-ScOpa09 | 28.6 | 7.0 | 26.5 | 4.8 | 20.0 | 6.3 | |  |
|  |  |  |  |  |  |  | |  |
| DD44-locus1* | 16.7 | 3.6 | 7.4 | 3.0 | 5.0 | 3.4 | |  |
| DD44-ScOpa09* | 33.3 | 7.3 | 8.1 | 3.3 | 12.8 | 5.4 | |  |
|  |  |  |  |  |  |  | |  |
| locus1-ScOpa09 | 9.1 | 4.3 | 15.7 | 4.0 | 14.2 | 5.4 | |  |
|  |  |  |  |  |  |  | |  |
| * data suggesting different position of DD44 in *S.latifolia and dioica.* | | | | | | |  | |
